# RNA Sequencing-Based Genome Reannotation of the Dermatophyte *Arthroderma benhamiae* and Characterization of Its Secretome and Whole Gene Expression Profile during Infection

**DOI:** 10.1128/mSystems.00036-16

**Published:** 2016-08-02

**Authors:** Van Du T. Tran, Niccolò De Coi, Marc Feuermann, Emanuel Schmid-Siegert, Elena-Tatiana Băguţ, Bernard Mignon, Patrice Waridel, Corinne Peter, Sylvain Pradervand, Marco Pagni, Michel Monod

**Affiliations:** a Vital-IT group, SIB Swiss Institute of Bioinformatics, Lausanne, Switzerland; b Department of Dermatology, Centre Hospitalier Universitaire Vaudois, Lausanne, Switzerland; c Swiss-Prot Group, SIB Swiss Institute of Bioinformatics, Geneva, Switzerland; d Fundamental and Applied Research for Animals & Health (FARAH), Department of Infectious and Parasitic Diseases, Faculty of Veterinary Medicine, University of Liège, Liège, Belgium; e Protein Analysis Facility, Center for Integrative Genomics, University of Lausanne, Lausanne, Switzerland; f Genomic Technologies Facility, Center for Integrative Genomics, University of Lausanne, Lausanne, Switzerland; University of Pittsburgh

**Keywords:** *Arthroderma benhamiae*, RNA-seq, *Trichophyton*, annotation, dermatophytes, infection, proteases, secreted proteins

## Abstract

Dermatophytes are the most common agents of superficial mycoses in humans and animals. The aim of the present investigation was to systematically identify the extracellular, possibly secreted, proteins that are putative virulence factors and antigenic molecules of dermatophytes. A complete gene expression profile of *Arthroderma benhamiae* was obtained during infection of its natural host (guinea pig) using RNA sequencing (RNA-seq) technology. This profile was completed with those of the fungus cultivated *in vitro* in two media containing either keratin or soy meal protein as the sole source of nitrogen and in Sabouraud medium. More than 60% of transcripts deduced from RNA-seq data differ from those previously deposited for *A. benhamiae*. Using these RNA-seq data along with an automatic gene annotation procedure, followed by manual curation, we produced a new annotation of the *A. benhamiae* genome. This annotation comprised 7,405 coding sequences (CDSs), among which only 2,662 were identical to the currently available annotation, 383 were newly identified, and 15 secreted proteins were manually corrected. The expression profile of genes encoding proteins with a signal peptide in infected guinea pigs was found to be very different from that during *in vitro* growth when using keratin as the substrate. Especially, the sets of the 12 most highly expressed genes encoding proteases with a signal sequence had only the putative vacuolar aspartic protease gene *PEP2* in common, during infection and in keratin medium. The most upregulated gene encoding a secreted protease during infection was that encoding subtilisin SUB6, which is a known major allergen in the related dermatophyte *Trichophyton rubrum*.

**IMPORTANCE** Dermatophytoses (ringworm, jock itch, athlete’s foot, and nail infections) are the most common fungal infections, but their virulence mechanisms are poorly understood. Combining transcriptomic data obtained from growth under various culture conditions with data obtained during infection led to a significantly improved genome annotation. About 65% of the protein-encoding genes predicted with our protocol did not match the existing annotation for *A. benhamiae*. Comparing gene expression during infection on guinea pigs with keratin degradation *in vitro*, which is supposed to mimic the host environment, revealed the critical importance of using real *in vivo* conditions for investigating virulence mechanisms. The analysis of genes expressed *in vivo*, encoding cell surface and secreted proteins, particularly proteases, led to the identification of new allergen and virulence factor candidates.

## Abstract (precis)

Dermatophytoses (ringworm, jock itch, athlete’s foot, and nail infections) are the most common fungal infections, but their virulence mechanisms are poorly understood. Combining transcriptomic data obtained from growth under various culture conditions with data obtained during infection led to a significantly improved genome annotation. About 65% of the protein-encoding genes predicted with our protocol did not match the existing annotation for *A. benhamiae*. Comparing gene expression during infection on guinea pigs with keratin degradation *in vitro*, which is supposed to mimic the host environment, revealed the critical importance of using real *in vivo* conditions for investigating virulence mechanisms. The analysis of genes expressed *in vivo*, encoding cell surface and secreted proteins, particularly proteases, led to the identification of new allergen and virulence factor candidates.

## INTRODUCTION

Pathogenic dermatophytes are the most common agents of superficial mycoses, almost exclusively infecting the stratum corneum, nails, and hair (1, 2). The genomes of these fungi, smaller than those of *Aspergillus* spp., range from 22.5 to 24 Mb and are highly collinear. The number of predicted protein-encoding genes varies from 7,980 in *Arthroderma benhamiae* to 8,915 in *Microsporum canis* (3, 4). A large number of orthologs were found to be shared by all dermatophytes (6,158 groups, including paralog duplications) (4). Dermatophyte genomes were found to be enriched in genes encoding secreted proteases and depleted in genes encoding enzymes involved in sugar metabolism, as for example those typically involved in plant cell wall breakdown. These differences from other fungi attest to the high specialization of dermatophytes and their adaptation to particular proteinaceous substrates other than vegetal debris.

The molecular mechanisms involved in the establishment of dermatophyte infections are poorly understood and remain an open field of investigation. Host-fungus interactions involve pathogen offense, host defense, and pathogen counterattack. In these processes, fungal and host cell-associated and secreted proteins play a major role. For instance, secreted aspartic proteases are now considered important virulence factors of *Candida albicans*, being associated with adhesion, invasion, and tissue damage (5). Secreted enzymes referred to as “effectors” are also of major importance for host attack by plant pathogens (6). Likewise, proteins secreted *in vivo*, in particular proteases, are clearly the best candidates for virulence factors of dermatophytes.

Current knowledge regarding dermatophyte gene expression during infection was acquired using a cDNA microarray based on transcripts of *A. benhamiae*, grown in a protein medium, covering approximately 20 to 25% of its genome and on a few selected protease-encoding genes (7). As a striking result, the genes encoding most major proteases secreted by the fungus *in vitro* (8–11) were found to be not expressed *in vivo*, and therefore, these proteases appeared not to be involved during the establishment of infection. In contrast, the gene encoding the subtilisin SUB6 was found to be highly expressed during skin infection but not when the fungus grew in any culture medium. Of particular importance, SUB6 is the ortholog of the major allergen Tri r2 in *Trichophyton rubrum* (12). Tri r2 was found to induce dual immune responses and elicit either immediate or delayed-type hypersensitivity skin test reactions in different individuals. Numerous antigenic molecules eliciting host immune responses still remain to be discovered. In view of the importance of secreted proteins, both as antigens and as possible virulence factors, the goals of this work were the following: (i) to obtain a complete gene expression profile of *A. benhamiae* during infection using state-of-the-art RNA sequencing (RNA-seq) technology, (ii) to compare it with the expression profiles of the fungus grown *in vitro* in different media, and (iii) to identify which proteins, and in particular individual proteases, are secreted *in vivo* during infection as possible new virulence factors. By exploiting RNA-seq data for *A. benhamiae* growing under different culture conditions and during infection in guinea pigs, we first established a new annotation of the genome, with 7,405 protein-encoding genes. The previously available genome annotation of *A. benhamiae* showed its limits, as many discrepancies were found after comparison with new experimental data.

## RESULTS

### *Arthroderma benhamiae* experimental infections in guinea pigs.

**FIG 1:**
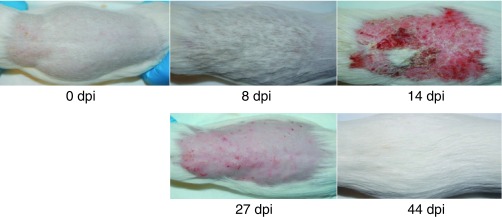
Experimental infection of the natural host of *Arthroderma benhamiae*. Cutaneously infected guinea pigs developed skin symptoms that were the most severe at 14 days postinfection (dpi) due to inflammation, while 8 dpi was the time point for the peak of infection.

Skin samples from experimentally infected animals were used for transcriptomic analysis of *A. benhamiae* during infection. As shown in Fig. 1, at day 8 after infection, the animals showed no or minimal skin symptoms. The direct mycological examination showed numerous filaments present on the hair and skin samples with the presence of a low number of conidia (data not shown). At 14 days, the guinea pigs exhibited macroscopic skin lesions, but direct mycological examination showed fewer fungal filaments on the infected skin samples with thicker septa than at 8 days. We considered day 8 as the time point for the peak of infection and day 14 as the time point for the peak of inflammation. After 27 days, the skin lesions were still present but regressing, while very few fungal elements were observed by direct mycological examination. At day 44, the guinea pigs had fully recovered from infection, and no *A. benhamiae* filaments were observable. At this time, three animals that had recovered from primary infection were reinfected by *A. benhamiae* but did not develop a new infection.

### RNA sequencing.

RNA was extracted in triplicate from the fungus grown in keratin medium, soy protein medium, and Sabouraud medium and from each infected animal. Approximately 13 million strand-specific reads were obtained for each RNA sample extracted from the fungus growing in the three tested culture media (Table 1). Approximately 30 million strand-specific reads were acquired from each RNA sample extracted from infected skin samples, consisting of a mixture of reads from the fungus and from its mammalian host. As a result, roughly 1 million fungal reads (2.8%) were obtained with RNA extracted from skin samples of guinea pigs at day 8 of infection, while 91.3% of the reads could be aligned with the guinea pig genome.

**TABLE 1:** RNA-seq data summary^a^

Library	Total no. of cleaned reads (M)	Reads aligned with organism:			
		*A. benhamiae*		*Cavia porcellus*	
		No. of reads	%	No. of reads (M)	%
8 dpi	34.5	0.5 M	1.5	31.8	92.3
	31.7	1 M	3.3	28.9	91.0
	26	1 M	4	23.5	90.6
14 dpi	31.8	44.5 K	0.1	30	94.3
	30.6	51.2 K	0.2	28.9	94.4
	31.4	24.8 K	0.1	29.5	93.8
27 dpi	33.8	623	0	31.6	93.5
	39	657	0	36.6	94.0
	30.8	452	0	28.8	93.3
44 dpi	35.7	458	0	33.1	92.9
	31.9	857	0	29.6	92.7
	25.3	808	0	23.5	92.8
Control	26.1	637	0	24.3	93.4
	38.9	840	0	36.3	93.3
	35.7	3,143	0	33.2	93.0
Keratin	12.4	6.1 M	49.2		
	13.5	7.9 M	58.3		
	13.9	8 M	57.6		
Soy	11.7	7.3 M	62.6		
	10.5	6 M	57.1		
	12.8	7.5 M	58.8		
Sabouraud	12.4	7.9 M	63.5		
	14.8	8.7 M	59.1		
	11.6	7.2 M	61.6		

a M, million; K, thousand; dpi, days postinfection.

### New gene annotation of the *Arthroderma benhamiae* genome.

A preliminary investigation of the RNA-seq reads mapped onto the *A. benhamiae* genome revealed that many gene and intron locations from the original genome annotations were not supported by our experimental data. Hence, reannotating the coding sequence (CDS) of the genome appeared to be a prerequisite before further analyzing the transcriptome expression. Particular attention was paid to the location of the start codons because of our interest in secreted proteins, which should be endowed with a signal peptide at the N terminus.

We used Augustus (13), a program for gene prediction in eukaryotic organisms that relies on a statistical model of an organism’s gene structure. The correctness of Augustus predictions is, however, highly dependent on this model, and great care must be used at the time of training this model (i.e., establishing the model using a training data set). Practically, we mapped all RNA-seq reads onto the genome, deduced full-length gene transcripts, and retained only those with sufficient coverage. Then, we translated the filtered transcripts into their three possible coding frames. Full-length CDSs were detected by aligning the transcripts against a set of high-quality protein sequences, namely, the protein sequences reviewed by Swiss-Prot of the model organisms *Saccharomyces cerevisiae* and *Aspergillus nidulans*. The CDS annotations were back-propagated onto the genome, introducing intron descriptions, and supplied as a training set to Augustus to generate a new gene model. With the latter, the *A. benhamiae* genome was reannotated and yielded 7,405 protein-encoding genes.

Table 2 compares our 7,405 newly predicted genes with the original set of 7,979 and shows that about 65% of the genes have been affected one way or another: for example, the intron boundaries within 1,246 genes were corrected and 383 new genes were recorded. In addition, 39 genes in the existing annotation were split into two genes, and, in contrast, 286 genes in the new annotation corresponded to fusions of previously annotated genes.

**TABLE 2:** Comparison of new gene set and original one^a^

New versus old gene prediction	Gene count in complete genome	Gene count in secretome only			
		With GPI		Without GPI	
		Auto	Manual	Auto	Manual
Matched	2,662	47 (13)	2 (2)	155 (55)	0
Alternative	1,246	19 (6)	0	49 (19)	1
Different	2,752	31 (6)	1	83 (19)	10 (4)
Merged	286	5 (2)	0	7 (2)	1
Split	76	1 (1)	0	5 (2)	0
New	383	6	0	34 (8)	0
Total	7,405	109 (28)	3 (2)	333 (105)	12 (4)

a Matched, identical old and new gene annotations; alternative, conserved start and stop codons but different splicing; different, different start or stop codons, possibly different splicing; merged, more than one old gene merged into a single new one; split, old gene split into several new ones; new, genes found only in the new predictions (708 original genes were lost); auto, gene annotations as produced by Augustus; manual, manual correction of the start codon. The number of genes whose products were confirmed by mass spectrometry in culture supernatants is given in parentheses. GPI, glycosylphosphatidylinositol.

### *In silico* definition of the secretome.

**FIG 2:**
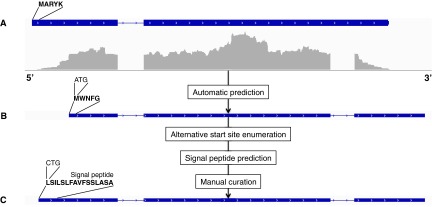
Prediction and manual correction of the gene coding for the autophagy protein Atg27 (ARB_01857; a transmembrane protein). (A) Original gene prediction; (B) automatic prediction from Augustus (signal peptide is missing); (C) final (new) gene prediction after manual correction. The reannotation of this particular gene is remarkable, as it produced a new intron, an alternative stop codon, and a manually corrected start codon.

We defined the secretome as the set of all secreted proteins, which is made of all proteins with a signal peptide, excluding transmembrane proteins. In practice, this set is not trivial to define. The presence/absence of a signal peptide depends on the tools used to predict it, on the strength of the signal itself, and on its presence at the N terminus, which ultimately relies on the correct detection of the start codon. Hence, all genes predicted by Augustus were further subjected to prediction refinements as follows. For every predicted CDS, variants were enumerated by considering every AUG or CUG (14) as an alternative start codon, when found within 30 amino acids from the AUG given by Augustus. Signal peptides were then searched for in all CDS variants. The retained CDS was finally selected manually by comparing the results of the different predictions and by considering additional evidence, such as prior biological knowledge or the presence of a glycosylphosphatidylinositol (GPI) anchor at the C terminus. GPI anchors affect the localization of these proteins in the plasma membrane or the cell wall, but removal of the GPI lipid moiety by phospholipases can generate soluble secreted forms of the protein (15). The overall procedure of gene prediction followed by manual correction is summarized with an example in Fig. 2.

A total of 634 proteins with a signal peptide, including 112 probable GPI-anchored proteins, has been predicted. Using transmembrane predictors, we removed all proteins that contained one or more transmembrane spans in addition to the signal peptide and that were probably targeted to membranes. This refinement led to a final *A. benhamiae* predicted secretome, made of 457 proteins that are listed and characterized in Table S1 in the supplemental material.

10.1128/mSystems.00036-16.1 Table S1  The secretome: predicted cell surface/secreted proteins, putative functions, and expression. ^1^Open reading frame (ORF) names in this study. ^2^The status indicates the changes between previous proteome annotation and our prediction. ^3^ORF names in the previous genome annotation (Burmester et al., 2011). ^4^Names attributed to some proteases by Burmester et al. (2011) and Sriranganadane et al. (2011). ^5^UniProt accession numbers corresponding to the previous prediction. When two ORFs have been merged in the new prediction and both are present in UniProtKB, the two corresponding ACs are indicated. ^6^The presence of a signal peptide is indicated by SIG. SIG+GPI indicates that the gene product is predicted to have a GPI anchor. ^7^Mass spectrometry data were extracted from the work of Sriranganadane et al. (2011). For each identified gene product, we indicate the medium pH in which it was detected (either pH 4 or 7). ^8^Function has been assigned based on homology search in well-characterized fungi and/or from InterPro scanning to identify specific domains and families. Green identifies proteins with a potential role in proteolytic activity; red, proteins involved in carbohydrate metabolism; and orange, proteins involved in lipid metabolism. ^9^Homologous fungal allergens extracted from the Allergome database (http://www.allergome.org/). ^10^Name of weighted gene correlation network analysis (WGCNA) gene coexpression module. ^11^Summary of differential gene expression *in vivo* versus *in vitro*. Cutoffs: FDR = 1e−3 and 2-fold change. ^12^Significant expression trends from RNA sequencing data are indicated. The cutoff of −1 for the *Z*-score of transcripts per million was applied. ^13^Mean expression values expressed in transcripts per million for every growth condition. The detailed counts per sample are given in Table S3. Download Table S1, XLSX file, 0.1 MB. Copyright © 2016 Tran et al. 2016 Tran et al. This content is distributed under the terms of the Creative Commons Attribution 4.0 International license.

**FIG 3:**
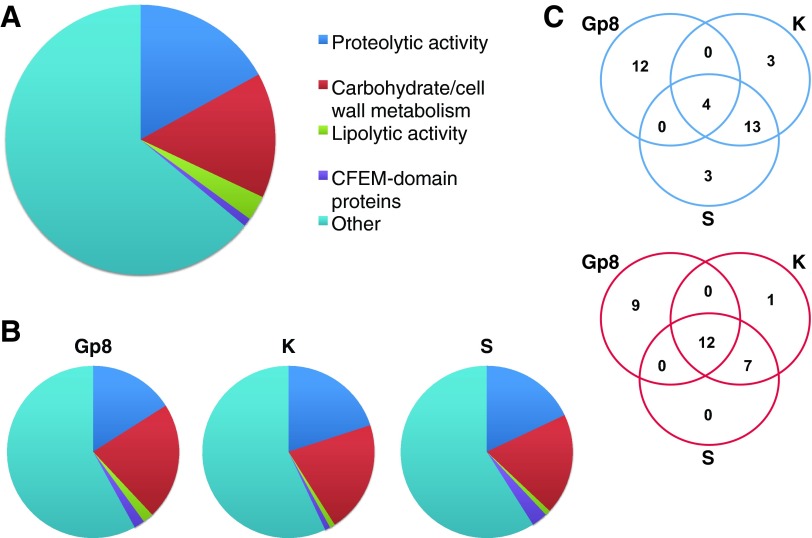
Characterization of the secretome. (A) Pie chart showing the main functional groups identified within the 457 proteins of the secretome. See detailed description in Data Set S1 in the supplemental material. (B) Pie charts showing the same functional groups as in panel A but within the 100 most expressed genes in Gp8 (*in vivo* 8 days postinfection), K (*in vitro* in keratin medium), and S (*in vitro* in soy medium). (C) Venn diagram of proteases (top) and carbohydrate/cell wall metabolism proteins (bottom) present in the 100 most expressed secreted proteins under the 3 conditions described for panel B. Proteases represent about 20% of the 100 most expressed proteins under the 3 conditions; however, the batch of proteins in Gp8 is clearly different from those in K and S. This trend is not as significant when comparing carbohydrate/cell wall metabolism proteins.

A few *A. benhamiae* proteins have been experimentally characterized, in particular secreted proteases (16) and hydrophobin HypA (17). In order to associate functional information with predicted proteins, we searched for homologs using Blast against UniProtKB (18), paying particular attention to the matches against *S. cerevisiae*, the best-characterized fungus; *C. albicans*, the best-characterized yeast pathogen; and filamentous fungi, such as *Aspergillus* spp. We completed functional predictions by checking for the presence of specific domains or protein family signatures by scanning the InterPro database (19, 20). We were able to associate putative functions with 316 out of our 457 predicted cell surface/secreted proteins, including main functional groups such as proteases, carbohydrate/cell wall metabolism proteins, or proteins with lipolytic activities (Fig. 3A; see also Data Set S1 in the supplemental material for details). In addition to thaumatin-like proteins, we identified 46 gene products showing homologies to known allergens (see Table S2 in the supplemental material), of which 21 were predicted to be secreted. Among the 141 uncharacterized secreted proteins, 25 had homologs in other dermatophytes, suggesting that they are involved in dermatophyte-specific functions/processes.

10.1128/mSystems.00036-16.10 Data Set S1  Supplemental materials and results. Download Data Set S1, PDF file, 0.2 MB. Copyright © 2016 Tran et al. 2016 Tran et al. This content is distributed under the terms of the Creative Commons Attribution 4.0 International license.

10.1128/mSystems.00036-16.2 Table S2  Potential allergens based on sequence homology. ^1^Open reading frame (ORF) names in this study that have homologs acting as allergens in other fungi (and wasp in the case of ARB_02861). ^2^Allergens were retrieved from the Allergome database (http://www.allergome.org/). ^3^The (+) indicates *A. benhamiae* allergen homologs identified as encoding putative cell surface/secreted proteins in our study. ^4^Function has been assigned based on homology search in well-characterized fungi and/or from InterPro scanning to identify specific domains and families. Download Table S2, XLSX file, 0.1 MB. Copyright © 2016 Tran et al. 2016 Tran et al. This content is distributed under the terms of the Creative Commons Attribution 4.0 International license.

### Validation of the new gene predictions of the secretome.

The secretome can be relatively easily subjected to investigation by mass spectrometry (MS) because it represents a small fraction of all proteins, and those found in the supernatant of *in vitro*-grown cultures can be recovered easily. We conducted a new analysis of the MS data that we previously published (16) regarding proteins secreted by cells grown in soy protein liquid medium, using the new secretome definition. The presence of 139 proteins in the supernatant at either pH 4 or 7 was confirmed (see Table S1 in the supplemental material), including 8 of the newly predicted ones. Moreover, among the 708 proteins from the original annotation that were lost in our new prediction, 31 were supposed to be secreted, but none of them could be detected in our MS data.

Similarity search is another way to test the quality of gene prediction. As an example, ARB_07403 encodes a putative A1 peptidase. In our prediction, ARB_07403 was shortened at the N terminus by 68 residues. This correction not only allows for the identification of a strong signal peptide at the new N terminus but also aligns better with the sequences of orthologs in closely related species, including TRV_06366 of *Trichophyton verrucosum* (UniProt accession no. D4DGR1) and MCYG_07979 of *Arthroderma otae* (UniProt accession no. C5FZ57).

However, it happens that neither prediction fitted with related proteins, requiring a further step of manual sequence correction. ARB_06467 (SUB10) and ARB_04678 (SED3) were found by similarity search to belong to the S8 and S53 families of serine proteases, respectively, but the predicted proteins missed the N-terminal signal peptide and propeptide. A reanalysis of the nucleotide sequence of SUB10 revealed a probable genome assembly error in a poly(T) stretch localized just behind the actual initiator codon, leading to a frameshift at position 4 (accession no. KX519317) (see Fig. S1A in the supplemental material). An error was also identified within the coding sequence of ARB_04677, upstream of ARB_04678. Correcting this error removed a frameshift at residue 109 of ARB_04677 and led to the fusion of the two open reading frames (ORFs) (accession no. KX519316) (see Fig. S1B). Sanger resequencing of the regions surrounding the two predicted errors confirmed our predictions and allowed us to restore both protease sequences with clear signals and propeptides. The actual protein sequences of SUB10 and SED3 have been updated in the UniProtKB database (accession numbers D4AQG0 and D4AK75, respectively).

10.1128/mSystems.00036-16.5 Figure S1  Manual curation on SUB10 and SED3*.* Actual and previous open reading frame predictions for SUB10 (A) and SED3 (B). N-terminal signal peptides, propeptides, and peptidase domains are illustrated with boxes of different hues. The corrections of assembly errors in actual open reading frames are indicated in red. Full ORFs were restored by the manual addition of the missing T in the SUB10 DNA sequence and G in the SED3 DNA sequence. These corrections have been confirmed by Sanger resequencing. The actual protein sequences of SUB10 and SED3 are available on the UniProtKB database (http://www.uniprot.org/) with respective accession numbers D4AQG0 and D4AK75. Download Figure S1, PDF file, 0.1 MB. Copyright © 2016 Tran et al. 2016 Tran et al. This content is distributed under the terms of the Creative Commons Attribution 4.0 International license.

Finally, it is interesting that, within the 40 new predicted ORFs, sequence alignments with other fungal proteomes revealed that two have homologs in filamentous fungi, such as *Aspergillus* species, and 22 are conserved in other dermatophyte species (see Table S1 in the supplemental material).

### *Arthroderma benhamiae* gene expression under different growth conditions.

**FIG 4:**
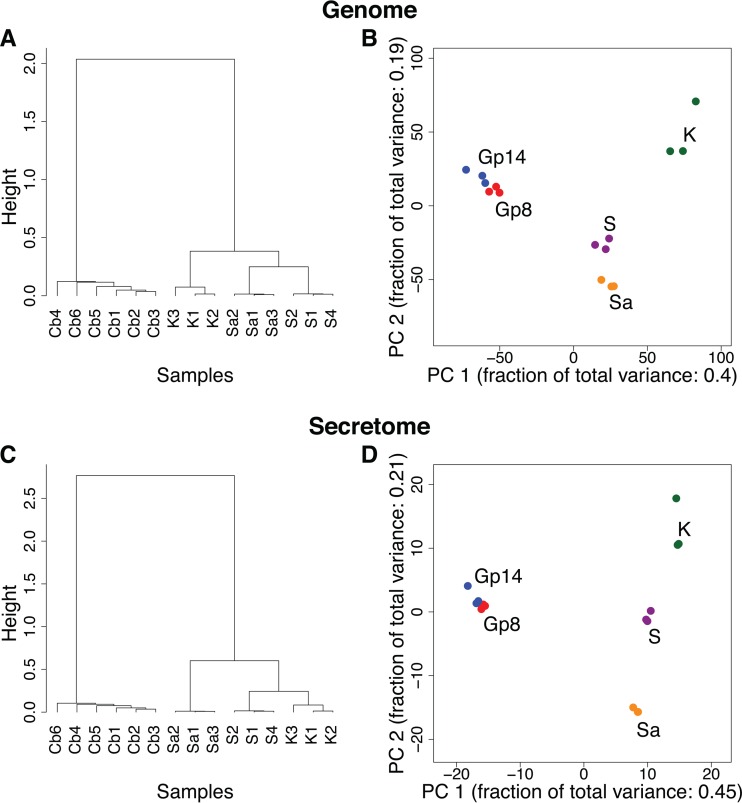
Hierarchical clustering (A and C) and principal component (PC) analysis (B and D) of RNA sequencing samples considering the genes from the complete genome (A and B) or only the secretome subset (C and D). The sample names reflect the growth conditions: Cb, *in vivo* in guinea pig; S, *in vitro* in soy medium; Sa, *in vitro* in Sabouraud medium; K, *in vitro* in keratin medium. The *in vivo* samples cluster together.

Gene expression levels were computed by mapping the reads onto the newly predicted gene set and are expressed as TMM-normalized Voom-transformed counts (see Table S3 in the supplemental material). The nomenclature used for the samples and the corresponding growth conditions are given in Table 3. Figure 4 presents an overview of the gene expression in the different samples, considering either the complete genome or the secretome subset. Both hierarchical clustering and principal component analysis indicate that the biological replicates are closer to each other than to other conditions, even for the *in vivo* samples at 14 days postinfection, where the number of obtained fungal reads (about 50,000) is possibly too low to perform a statistically significant analysis. However, the small distinction between the Gp8 and Gp14 *in vivo* conditions, which is on the order of intra-Gp variations, seems to indicate the consistency of Gp14 samples.

10.1128/mSystems.00036-16.3 Table S3  Detailed counts per sample, differential expression, and weighted gene correlation network analysis module attribution. Download Table S3, XLSX file, 1.4 MB. Copyright © 2016 Tran et al. 2016 Tran et al. This content is distributed under the terms of the Creative Commons Attribution 4.0 International license.

**TABLE 3:** Designation of samples and growth conditions

RNA sample	Growth condition	
	Code	Description
Cb1	Gp8	*In vivo*: guinea pig 8 days postinfection
Cb2		
Cb3		
Cb4	Gp14	*In vivo*: guinea pig 14 days postinfection
Cb5		
Cb6		
K1	K	*In vitro*: keratin medium
K2		
K3		
S1	S	*In vitro*: soy medium
S2		
S4		
Sa1	Sa	*In vitro*: Sabouraud medium
Sa2		
Sa3		

The expression differences are strongly dominated by the contrast between *in vivo* Gp8+Gp14 and *in vitro* S+Sa+K conditions. This result confirms and generalizes the observations made previously on a much smaller gene set (7). The analysis of the expression data from the complete genome (including the secretome) and of the secretome yielded the same strong contrast, possibly even slightly reinforced for the secretome.

**FIG 5:**
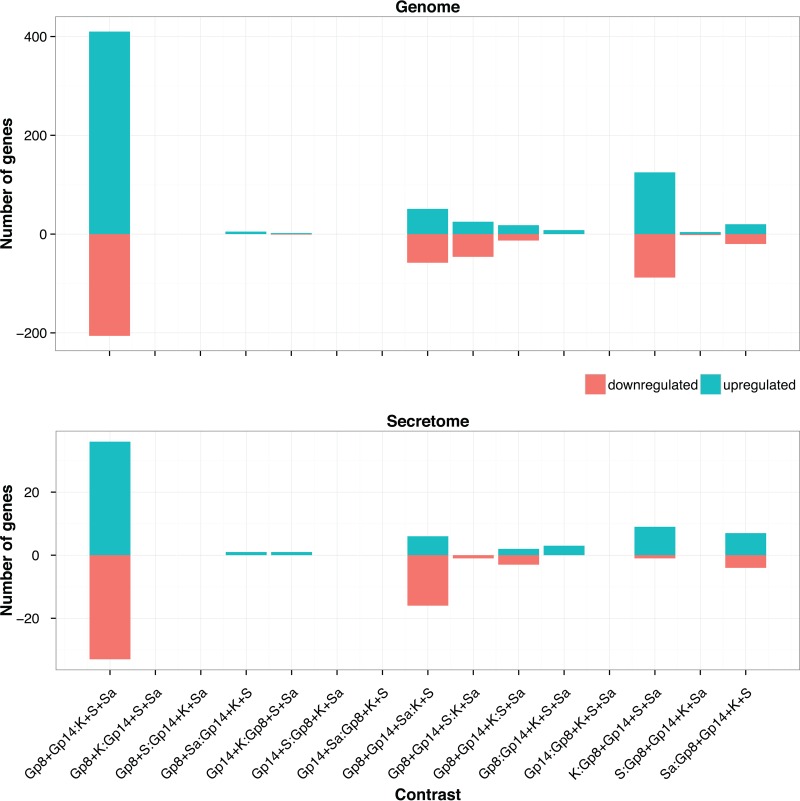
Number of differentially expressed genes versus the enumeration of all possible contrasting conditions in the genome and the secretome**,** using a cutoff of 1e−3 for FDR and 2 for the fold change.

Among the *in vitro* conditions, the gene expression levels in the soy and Sabouraud media appeared closer to each other in the complete gene set, while soy and keratin appeared closer in the secretome subset. None of the three *in vitro* conditions tested is a good proxy for *in vivo* growth conditions, despite the keratin medium being supposed to mimic the host environment. To address this question in more depth, we enumerated all possible partitions of growth conditions into two subsets, to contrast a subset of conditions with the remaining ones. The list of all possible contrasts is given in Fig. 5, with the corresponding amounts of differentially expressed genes. This confirms that the *in vivo*-*in vitro* contrast is dominant and that not much information can be expected to be gathered by separating Gp8 from Gp14. Interestingly, two other contrasts seem to carry additional signals: K:Gp8+Gp14+Sa+S in the genome complete gene set and Gp8+Gp14+Sa:S+K in the secretome subset.

We utilized a different statistical approach, namely, weighted gene correlation network analysis (WGCNA) and gene ontology (GO) enrichment analysis, to further explore these additional contrasts. The unsupervised clustering algorithm of WGCNA subdivided the input gene set (genome) into 35 different modules, which are disjoint subsets of genes. Then, these modules were individually correlated with the 15 possible different contrasts to detect optimal correlations. As shown in Fig. S2 in the supplemental material, the *in vivo*-*in vitro* contrast again dominated the results, with 2,122 genes found in the turquoise and blue modules. Figure S3A in the supplemental material presents the gene expression heat map for the turquoise module as an example. The blue module also showed a high correlation with the *in vivo*-*in vitro* contrast, although the expression in Sabouraud medium was intermediate (see Fig. S3B). A few other smaller modules appeared to be correlated with different contrasts, such as the tan module with 209 genes that strongly correlates with K:Gp8+Gp14+Sa+S (see Fig. S3C) and the midnight blue module with 177 genes, highly correlating with Gp8+Gp14+S:K+Sa (see Fig. S3D). The 323 genes from the yellow module also correlate with Gp8+Gp14+S:K+Sa, despite an intermediate expression in Sabouraud medium (see Fig. S3E).

10.1128/mSystems.00036-16.6 Figure S2  Module-contrast correlations. Download Figure S2, PDF file, 0.1 MB. Copyright © 2016 Tran et al. 2016 Tran et al. This content is distributed under the terms of the Creative Commons Attribution 4.0 International license.

10.1128/mSystems.00036-16.7 Figure S3  Heat map of the turquoise module (A), blue module (B), tan module (C), midnight blue module (D), and yellow module (E). Download Figure S3, PDF file, 1.1 MB. Copyright © 2016 Tran et al. 2016 Tran et al. This content is distributed under the terms of the Creative Commons Attribution 4.0 International license.

We mapped about 40% of the predicted proteins of *A. benhamiae* to their orthologous counterparts in *S. cerevisiae* using Inparanoid and propagated the latter gene ontology (GO) annotations onto the dermatophyte genes. Table S4 in the supplemental material presents the modules for which the most significant GO term enrichment was detected, especially the yellow, midnight blue (correlated with K+Sa:Gp8+Gp14+S), and tan (correlated with K:Gp8+Gp14+Sa+S) modules. The results are, however, very general, revealing changes in translational and RNA-related activities but also indicating that some proteasome-related activities might be specifically altered during growth on keratin. These somewhat modest results are certainly more related to the lack of specific gene annotation for *A. benhamiae* than to a lack of well-formed gene modules.

10.1128/mSystems.00036-16.4 Table S4  Pathway enrichment analysis of weighted gene correlation network analysis modules. Only the most significant gene ontology terms are reported. Download Table S4, XLSX file, 0.1 MB. Copyright © 2016 Tran et al. 2016 Tran et al. This content is distributed under the terms of the Creative Commons Attribution 4.0 International license.

### Gene expression profile of *Arthroderma benhamiae* cell surface/secreted proteins during inflammatory cutaneous infection highly differs from the profile obtained during growth on keratin.

**FIG 6:**
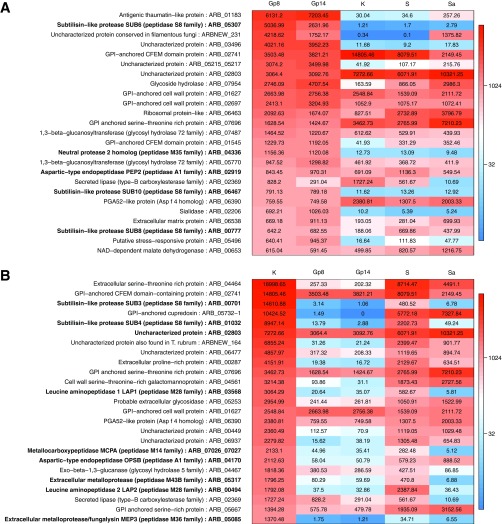
(A) The twenty-five most highly expressed genes encoding secreted proteins during infection compared to *in vitro* expression. (B) The twenty-five most highly expressed genes encoding secreted proteins *in vitro* (keratin medium) compared to *in vivo* expression. Abbreviations are as defined in the Fig. 4 legend.

Figure 6A lists the 25 secretome genes most highly expressed *in vivo*, including five putative protease genes. The first gene, ARB_01183, encodes a protein which contains a thaumatin domain. The second gene, ARB_05307, encodes the subtilisin SUB6. Four genes encode proteins for which we did not find any functional data. These include ARBNEW_231, a newly predicted gene and the third most highly expressed gene *in vivo.* Remarkably, the secretome expression pattern was completely different during growth on keratin, an *in vitro* condition that was supposed to mimic the host environment (Fig. 3B and C and 6B). Only five genes were found to be common to Fig. 6A and B: two encoding putative GPI-anchored proteins (ARB_01627 and ARB_07696), ARB_02741 encoding a CFEM domain protein, ARB_06390 encoding a putative cell wall protein, and ARB_02369 encoding a carboxylesterase domain-containing protein. This difference is even more striking when we focus our analysis on secreted proteases. Even if about 20% of the 100 most expressed secreted proteins are proteases both *in vivo* and in keratin (Fig. 3B), the batch of proteins expressed under these different conditions is clearly different (Fig. 3C). This is in accordance with our above-mentioned WGCNA in which relevant correlation groups were found only when *in vivo* and keratin conditions were contrasted (Gp8+Gp14:Sa+S+K, Gp8+Gp14+Sa:S+K, Gp8+Gp14+S:Sa+K, or Gp8+Gp14+Sa+S:K). Expression patterns in soy and Sabouraud media are closer to that in keratin, and yet they are distinct from each other (Fig. 4), which explains their relatively neutral impact in the WGCNA.

Figure S4 in the supplemental material lists the 12 most highly expressed genes encoding proteases during infection and those expressed on keratin. The genes encoding SUB6 (ARB_05307), SUB10 (ARB_06467), and the deuterolysin (ARB_04336) are highly and specifically upregulated during the infection phase with changes of 2,000-fold, 60-fold, and 100-fold, respectively. The gene encoding SUB8 (ARB_00777) was relatively downregulated in keratin. PEP2 (ARB_02919), which is a putative ortholog of the vacuolar aspartic protease of *S. cerevisiae* PrA and has been subsequently identified in other filamentous fungi, was found to be highly expressed under all the *in vivo* and *in vitro* conditions. On the other hand, the protease genes upregulated in keratin include subtilisins SUB3 (encoded by ARB_00701) and SUB4 (ARB_01032), the metallocarboxypeptidase MCPA of the M14 family (ARB_07026_07027), the leucine aminopeptidases LAP1 (ARB_03568) and LAP2 (ARB_00494), the aspartic protease OPSB (ARB_04170), and two extracellular metalloproteases (ARB_05085 and ARB_05317). Likewise, in the soy culture, only four protease genes were highly expressed: those for SUB4 (encoded by ARB_01032), LAP2 (ARB_00494), PEP2 (ARB_02919), and DPPV (ARB_06651) (see Table S1 in the supplemental material). With the Sabouraud culture, in addition to PEP2, SUB8 (ARB_00777), OPSB (ARB_04170), DPPIV (ARB_06110), and a gene encoding an uncharacterized S10 family protease (ARB_01491) showed relatively high expression (see Table S1).

10.1128/mSystems.00036-16.8 Figure S4  The twelve most highly expressed genes encoding secreted proteases during infection (left table) and during *in vitro* growth in keratin medium (right table). Download Figure S4, PDF file, 0.1 MB. Copyright © 2016 Tran et al. 2016 Tran et al. This content is distributed under the terms of the Creative Commons Attribution 4.0 International license.

10.1128/mSystems.00036-16.9 Figure S5  Quality of RNA extracted using the Qiagen RNA extraction kit (A) and the protocol described in Materials and Methods (B). The 18S rRNA and 28S rRNA absorbance peaks are shown in purple and blue, respectively. Download Figure S5, PDF file, 0.4 MB. Copyright © 2016 Tran et al. 2016 Tran et al. This content is distributed under the terms of the Creative Commons Attribution 4.0 International license.

## DISCUSSION

Most previously available dermatophyte ORFs had been deduced by cDNA analysis and by expressed sequence tag sequencing using RNA extracted from dermatophytes grown *in vitro*. RNA-seq data obtained from *A. benhamiae* grown under various liquid culture conditions and, most importantly, during infection in guinea pigs led us to an improved gene prediction and annotation of its genome. A complete gene expression profile of *A. benhamiae* was obtained during infection of its natural host.

### New *Arthroderma benhamiae* gene annotation.

About 65% difference and, particularly, 383 new protein-encoding genes were detected compared to the existing gene prediction. We used previously acquired MS data to validate *a posteriori* the presence of the predicted ORFs in culture supernatant. A comparable approach with emphasis on proteogenomics has been recently used to review the genome and proteome of *T. rubrum* (21). In this study, the identification of 323 new peptides by MS in culture supernatant led to the refinement of 161 genes and the prediction of nine new genes. However, the RNA-seq analysis to validate the whole-genome proteomics was performed only with RNA extracted from *T. rubrum* cultured *in vitro* on potato glucose agar but not during infection. This previous study and our results have in common the combination of experimental data with bioinformatics software and manual curation to generate an improved gene annotation. Our study focuses, furthermore, on the biology of infection.

*In silico* analysis of our predicted proteome led to the identification of 457 putative cell surface and secreted proteins. Our list of probable secreted proteins is likely to also contain proteins targeted to intracellular organelles, such as the endoplasmic reticulum or vacuole, since the exploited prediction tools cannot distinguish between such proteins and secreted ones. The Fungal Secretome and Subcellular Proteome KnowledgeBase (http://bioinformatics.ysu.edu/secretomes/fungi2/index.php) tries to address this concern by providing the prediction of secreted and organellar localization of proteins. It basically utilizes the same tools as those that we used in our strategy and reveals the same functional groups (22). In addition, they use WoLF PSORT (http://www.genscript.com/wolf-psort.html), which converts protein sequences into numerical localization features, based on sorting signals, amino acid composition, and functional motifs. Nevertheless, this tool can produce a high number of false positives. Moreover, homologs of well-known intracellular proteins have been found in the secretome proteomic data. As an example, ARB_02919 is the closest *A. benhamiae* homolog of the *A. fumigatus* vacuolar aspartic peptidase (PEP2) and *S. cerevisiae* vacuolar proteinase A (PEP4). The latter is a vacuolar enzyme required for the processing of vacuolar precursors (23), whereas the former plays an additional role linked to the cell wall (24). ARB_02919 was found as a secreted protein by MS (16) and is one of the most expressed proteins under all of the five studied conditions. Contaminations cannot be ruled out, but our strategy ensures the best coverage of cell surface and secreted proteins, even if some false positives are probably still present.

### Reprogramming of gene expression from a saprophyte to a parasite lifestyle.

Striking differences were revealed between transcriptomes of *A. benhamiae* during growth under various conditions *in vitro* and during infection of its natural host. Such differences emphasize the importance of performing transcriptional analysis directly during infection, instead of using *in vitro* conditions that are expected to mimic the host environment. We also identified several newly predicted genes, as well as genes with unknown functions, that were differentially expressed in the contrast of *in vivo* and *in vitro* and, thus, might have a relevant role in infection. To sum up, the ability of dermatophytes to switch from a saprophyte to a parasite lifestyle is attested by an important reprogramming of gene expression.

Several comparative RNA-seq analyses were performed for other species of human-pathogenic fungi (25–28), but as these studies rely on infection-mimicking conditions and not on the real *in vivo* situation, we think that they should be considered with caution. Only a few studies were performed under real infection conditions. Gene expression profiles of *C. albicans* were obtained during infection in both the mouse kidney and the insect *Galleria mellonella* (29). Interestingly, gene expression values in these very distinct hosts were much closer to each other than in the *in vitro* liquid cultures used as controls. More recently, transcriptional profiling of *Blastomyces* was performed in cocultures with human bone marrow-derived macrophages and during *in vivo* pulmonary infection in a mouse model (30). The authors identified a number of functional categories upregulated exclusively *in vivo*, including secreted proteins and zinc acquisition proteins, as well as cysteine and tryptophan metabolism. Nine secreted proteins were identified, including products of five of the 10 most upregulated genes during infection. One of these genes, BDFG_00717, encodes a CFEM domain-containing protein, highlighting the importance of those proteins in virulence.

### Potential nonprotease virulence factors of *Arthroderma benhamiae.*

Numerous genes that were highly expressed during infection encode uncharacterized proteins. Highly expressed protein-encoding genes with a putative function other than proteolysis included ARB_01183, encoding a putative antigenic thaumatin domain protein, and two genes encoding 1,3-beta-glucanosyltransferases (ARB_07487 and ARB_05770). ARB_01183 was the most highly expressed secreted protein-encoding gene *in vivo*. Thaumatin-like proteins (TLPs) are found in many eukaryotes and have been particularly studied in plants, in which they are involved in defense against fungal pathogens. Plant TLPs also have been shown to act as important allergens (31). TLPs are also found in fungi, such as *Moniliophthora perniciosa*, and may be involved in the inhibition of growth of fungal competitors and pathogenicity (32). The 1,3-beta-glucanosyltransferases play an important role in fungal cell wall morphology and pathogenicity. Deletion of the gene *GEL*2 encoding a 1,3-beta-glucanosyltransferase in *A. fumigatus* leads to altered cell wall composition as well as to reduced virulence in a murine model of invasive aspergillosis (33). GAS1 of the entomopathogenic fungus *Beauveria bassiana* contributes similarly to its mycoinsecticide activity (34).

ARB_02741, like *Blastomyces* BDFG_00717, encodes a GPI-anchored CFEM domain protein which is highly expressed under *in vivo* and *in vitro* conditions. Its function has not been characterized yet, but it is interesting that the closest homologs of ARB_02741 in the human fungal pathogen *Coccidioides posadasii* are the proline-rich antigens Ag2/PRA and Prp2, which have been reported to be leading vaccine candidates (35, 36). CFEM domain proteins have been shown to be important for heme uptake and virulence in *C. albicans* (37). The ability to acquire iron from host tissues is a major virulence factor of pathogenic microorganisms. However, the exact involvement of these proteins in infection processes is still unclear. As an example, the three *A. fumigatus* CFEM domain proteins have been shown to be important for cell wall stability, not for virulence (38). Other proteins may also be involved in immune escape, such as ARB_06975, whose *A. fumigatus* hydrophobin homolog was shown to prevent immune recognition by forming a hydrophobic layer on the cell surface (39).

### *Arthroderma benhamiae* secreted proteases during infection.

*SUB6* was the most highly expressed gene encoding a secreted protease during infection in guinea pigs. In addition to *SUB6*, other *A. benhamiae* protease genes, encoding the subtilisins SUB7, SUB8, and SUB10 as well as a neutral protease of the deuterolysin family (M35), were also specifically upregulated. RNA-seq analysis results also confirmed that genes encoding major proteases secreted by the fungus during growth in a protein medium (i.e., SUB3, SUB4, MEP3, MEP4, LAP1, and DPPIV) were expressed at a relatively low level during infection as well as in Sabouraud medium and were not upregulated. These results are in accordance with recent findings by proteomic analysis (liquid chromatography-tandem MS [LC-MS/MS]) in *T. rubrum*-infected nails that revealed SUB6 as the major protein secreted by the fungus in onychomycosis (40). The closely related SUB7 (subtilisin-like protease 7, Q8NID9) and DPPV (dipeptidyl-peptidase 5, Q9UW98) were also detected. Likewise, most major proteases secreted by the fungus during its growth *in vitro* in a protein medium (11, 41) were not detected and, therefore, appeared not to be involved during the establishment of onychomycosis. As a general conclusion, the proteases secreted *in vitro* during protein degradation and *in vivo* during infection are different, regardless of the dermatophyte species and the tinea. The view that the proteases isolated from dermatophytes grown *in vitro* in a protein medium are virulence attributes and exert a major role during infection appears to be too naive and can no longer be accepted. Dermatophytes evolved from soil saprophytic fungi that are able to efficiently degrade hard keratin into amino acids and into short peptides in the process of recycling nitrogen, and the pathogenic phase of dermatophytes has to be dissociated from their saprophytic phase. Some of the multiple members of protease gene families in dermatophytes are dedicated exclusively to protein degradation, while others, such as SUB6, likely fulfill specific roles during infection. The notion that proteases secreted in proteinaceous media correspond to virulence attributes has also been discarded for other pathogenic fungi. Two different *A. fumigatus* mutants unable to secrete proteolytic activity in a protein growth medium did not show attenuated virulence when tested in a leukopenic mouse model. In the first mutant, the genes encoding the two major secreted proteases ALP and MEP (42) were deleted. In the other mutant, the gene encoding a transcriptional activator (PRTT) which regulates transcription of genes encoding the major proteases secreted in a protein medium was deleted. Noteworthily, no homolog of PRTT in *Aspergillus* spp. (43, 44) has been identified in *A. benhamiae*.

Genes encoding major proteases secreted by dermatophytes during *in vitro* growth in a protein medium are tightly controlled by *DNR1*, the ortholog of *AREA* in *Aspergillus nidulans* (45). In the absence of ammonium and glutamine, this transcription factor was found to be required for the expression of genes involved in nitrogen metabolism. Although dermatophytes infect keratinized tissues, our results suggest that the panel of proteases secreted during infection depends on other transcription factors that remain to be discovered.

### *Arthroderma benhamiae* secreted proteins as allergens.

Secreted proteins are allergens that play a key role in the pathogenic process. SUB6, DPPV, and the beta-glucosidase ARB_05770 (encoded by three of the most highly expressed genes of *A. benhamiae* during infection) are orthologs of the three known major dermatophyte allergens Tri t1, Tri r2, and Tri r4, which are involved in bronchial sensitization and symptomatic asthma (12, 46, 47). Dermatophyte antigens are also involved in eczematous skin reactions at a location distant from the area of dermatophyte infection (dermatophytids). The etiology of common dyshidrotic and vesicular eczema on the hands (palms and fingers) is rarely investigated and may remain elusive because no commercially standardized antigens are available to perform routine skin tests and antibody detection. Trichophytin, a fungal extract that greatly varies in its preparation and composition, was used to diagnose dermatophytids (48, 49). The secreted proteins encoded by genes highly expressed during infection are the best candidates for the detection of dermatophyte allergic diseases. At a time when quality in laboratory techniques is a key issue, it would be relevant to perform skin test reactions using standardized antigens in cases of eczematous skin reactions of unknown origin. A positive reaction could be indicative of a nondetected dermatophyte infection and could suggest possible antifungal treatment.

### Conclusion.

Comparing gene expression during infection phase with keratin degradation *in vitro* shows the importance of using real *in vivo* conditions to further investigate the virulence mechanisms of dermatophytes, instead of using some *in vitro* conditions supposed to mimic the host environment. Focusing our analysis on genes encoding cell-associated and secreted proteins, in particular proteases, led to the identification of strong candidates as allergens and putative virulence factors. The new genome annotation provided in this study might serve as a reference for annotation or reannotation of other dermatophyte species and evolutionarily related filamentous fungi.

## MATERIALS AND METHODS

### Strains and growth media.

*Arthroderma benhamiae* Lau2354-2 (CBS 112371) (3, 50) was used in this study. This strain, deposited in the Belgian Coordinated Collections of Microorganisms (BCCM/IHEM) under IHEM20161, is the reference strain that was chosen for *A. benhamiae* genome sequencing (3). It was isolated from a patient suffering from a highly inflammatory dermatophytosis in the Centre Hospitalier Universitaire Vaudois (CHUV). The *A. benhamiae* strain was maintained at 28°C on Sabouraud dextrose agar medium.

*Arthroderma benhamiae* was grown *in vitro* in Sabouraud liquid medium, soy protein liquid medium, and keratin liquid medium as previously described (7). Soy medium was prepared by dissolving 2 g of soy protein (Supro 1711; Protein Technologies International) in 1 liter of distilled water. Aliquots of 100 ml of keratin medium were prepared by adding 0.2 g of keratin (Merck, Darmstadt, Germany; keratin is derived from animal hooves and horns) and 5 ml of soy medium to 95 ml of distilled water. A small amount of soy protein in keratin liquid medium was found to be necessary to initiate the growth of dermatophytes with keratin as the sole substrate (7). A plug of fresh *A. benhamiae* mycelium grown on Sabouraud agar was inoculated in 100 ml of liquid Sabouraud, soy, and keratin medium and incubated for 5, 10, and 24 days, respectively, at 30°C without shaking. At the indicated time points, growth in protein medium was accompanied by substantial proteolytic activity along with clarification of the medium and, in the case of keratin medium cultures, also by visible dissolution of the water-insoluble keratin granules.

### Animal infection.

Specific-pathogen-free, 3-month-old female guinea pigs (cross-bred white albinos, Dunkin Hartley strain; Charles River Laboratories International, Wilmington, MA, USA) were infected with *A. benhamiae* Lau2354-2. *Arthroderma benhamiae* mycelium scraped from freshly grown 18-day-old Sabouraud plates and suspended in 5% (wt/wt) poloxamer 407 (BASF, Germany) was applied to a 16-cm^2^ back skin surface that had been clipped and scarified previously. Each guinea pig was infected with 6 × 10^9^ to 2 × 10^10^ CFU. Noninfected control guinea pigs were subjected to the same procedure, except that the poloxamer 407 mixture did not contain any fungal elements. Three guinea pigs were sacrificed after 8, 14, 27, and 44 days and at 14 days after reinfection once they had healed. The infected skin from sacrificed animals was frozen at −80°C for subsequent total RNA isolation. Both the hair and stratum corneum were examined for the presence of fungal elements by direct mycological examination. Animal experiments were approved by the local ethics committee (University of Liège, ethics protocol no. 1052).

### RNA extraction.

RNA extraction from *A. benhamiae* cultures and infected guinea pig skin was performed using a specific procedure to yield sufficient amounts of quality RNA (see Data Set S1 in the supplemental material).

### RNA sequencing.

In close collaboration with the Lausanne Genomic Technologies Facility and using the Illumina technology (HiSeq 2000 sequencer), we performed a TruSeq stranded single read total RNA analysis, using one lane with a multiplex level of 15, acquiring approximately 30 million “strand-specific” reads with a length of 100 bp for each sample. Reads were aligned against the *A. benhamiae* and guinea pig genomes using tophat2 (version 2.0.9) (51).

### Strain.

The genome assembly GCA_000151125.2 ASM15112v2 of *A. benhamiae* Lau2354-2 was used throughout this study.

### Gene prediction and annotation.

Gene prediction was made with Augustus (version 3.0.2) (13) using a specific gene model obtained as follows. Gene transcripts and intron locations were obtained using Cufflinks (version 2.2.1) (52). The transcripts were three-frame translated into potential amino acid sequences using Transeq from EMBOSS (version 6.5.7) (53). The complete proteomes of *Saccharomyces cerevisiae* and *Aspergillus nidulans* (reviewed by Swiss-Prot) were mapped onto the potential amino acid sequences with Glsearch36, from the FASTA alignment tools (version 3.6) (54), to identify coding phase and CDS location within transcripts. Based on the alignment quality and on the presence of start and stop codons near alignment extremities (±10 amino acids), a set of confidently predicted CDSs was gathered and converted into gene annotations using intron locations previously given by Cufflinks. These annotations were used as a training set to build a gene model (available upon request) with the scripts supplied in the Augustus distribution.

### *In silico* identification of putative cell surface and secreted proteases.

To identify putative secreted proteins, we checked for the presence of an N-terminal signal sequence using both Phobius (version 1.01) (55) and SignalP (version 4.1) (56). Signal peptides have been confirmed by the prediction of N-terminal transmembrane spans using TMHMM (version 2.0) (57, 58). The presence of a potential glycosylphosphatidylinositol (GPI) anchor has been checked by using PredGPI (version 1.0) (59). Using the transmembrane span predictors TMHMM (version 2.0), ESKW (version 1.0) (60), and MEMSAT (version 1.8) (61), we refined the secretome prediction by removing the proteins that contain one or more transmembrane spans in addition to the signal peptide and that are probably targeted to membranes. All the secreted proteins have been subjected to Blast analysis against the UniProtKB database (18) as well as to InterPro scanning (19, 20) to associate and reveal some putative functions.

### Mass spectrometry and experimental validation of new secreted proteins.

Precipitation and separation of proteins from *A. benhamiae* cultures at pH 4 and pH 7 along with shotgun mass spectrometry (MS) experiments have been described by Sriranganadane et al. (16). A new search of MS/MS spectra against the sequences of our new predicted proteome was performed.

### Transcriptome analysis.

The number of reads mapped onto each newly predicted gene locus was obtained with Htseq-count (version 0.5.4p3) (62). Genes with counts of fewer than one per million in all samples were removed from the statistical analyses (i.e., 81 genes). Gene expression was normalized using the TMM-normalized Voom transformation (63); hierarchical clustering and principal component analysis were done using R (version 3.1.1). Differential gene expression analysis was performed with the R Bioconductor package Limma (64). The cutoffs of 1e−3 for false discovery rate (FDR) (Benjamini-Yekutieli-adjusted *P* value) (65) and 2 for fold change were applied to identify genes relevant to each contrast. The R software package WGCNA (66) was used for correlation network analysis, using the Pearson correlation.

### Pathway enrichment.

The predicted *A. benhamiae* proteins were aligned against *Saccharomyces cerevisiae* proteins from Swiss-Prot with Inparanoid (version 4.1) (67) to identify the orthologs from which the gene ontology (GO) terms were extracted and applied to *A. benhamiae*. We then performed the GO enrichment analysis on the weighted gene correlation network analysis (WGCNA) gene modules.

### Accession number(s).

The raw RNA-seq data investigated here are accessible under the SRA accession number SRP064455. The annotation has been deposited as a Whole Genome Shotgun project at DDBJ/ENA/GenBank under the accession number DAAX00000000. The version described in this paper is version DAAX01000000. The CavPor3 draft assembly of the guinea pig genome was used.
