# Supplementary material for: RNA Sequencing-Based Genome Reannotation of the Dermatophyte Arthroderma benhamiae and Characterization of Its Secretome and Whole Gene Expression Profile during Infection
Source: mSystems. 2016 Aug 2;1(4):e00036-16. doi: 10.1128/mSystems.00036-16 (PMC5069957; doi:10.1128/mSystems.00036-16)
Supplement: Data Set S1 [file sys004162042s1.pdf]

SUPPLEMENTARY MATERIALS

RNA extraction

A large amount of high-quality RNA could be extracted from *A. benhamiae* mycelia grown in liquid cultures by use of the QIAGEN© RNeasy Plant Mini Kit following the instructions of the manufacturer. However, the QIAGEN© protocol of RNA extraction was not suitable for extracting total RNA from infected guinea pig skin. The quality of the RNA extracted from infected guinea pig skin had a RNA quality number (RQN) of 2–4, which is sub-optimal for cDNA library construction and subsequent RNA-seq analysis. Therefore, two modifications were made to the extraction protocol, as described in the Materials and Methods section, to increase the RNA quality from the *in vivo* samples: (i) frozen pieces of collected guinea pig skin scrapings were mechanically broken with glass beads in TRIzol, and (ii) RNAsin was used in the final steps of extractions to stabilize and increase the quality of extracted RNA). This protocol was also used for RNA extraction from *A. benhamiae* mycelia grown *in vitro*.

The optimized protocol to extract RNA from skin of infected guinea-pigs was established as following: The fungal mycelia or guinea pig skin infected by *A. benhamiae* was frozen in liquid nitrogen and then crushed in a grinder in the presence of glass beads and phenol/guanidine isothiocyanate (TRIzol, Life Technology, Carlsbad, USA) to avoid RNA degradation. The resulting powder (250 µL) was placed in an Eppendorf tube with approximately 700 µL of glass beads and 500 µL of TRIzol. The fungal elements were mechanically broken down further using a FastPrep®-24 homogenizer (MP Biomedicals, LLC) for 15 s at a speed of 4 m/s, and then immediately put on ice. Next, 250 µL of phenol:chloroform:isomyl alcohol (24:24:1) (Life

Technologies) were added to the whole mixture. After vigorous mixing, a centrifugation step was performed for 6 min at 13000 rpm (19000 g). Two hundred microlitres of the aqueous phase were extracted and added to the same volume of 80% ethanol, mixed by pipetting, and then added to a RNeasy Mini Spin Columns (QIAGEN©, Venlo, Netherlands). After 15 s of centrifugation, the collector tube was removed (for the entire protocol after each centrifugation, the collector tubes were replaced), and then 350 µL of wash buffer RW1 (QIAGEN©) was added and the column centrifuged for 15 s. Subsequently, one unit of RNasin© Plus RNase inhibitor (Promega) in 20 µL of water was added, and the column was left for 5 min at room temperature. The column was then washed again with 350 µL of RW1 and centrifuged for 15 s.

In a next step, a solution of RNAfree DNase (QIAGEN©) (10 µL of DNase in 70 µL of RNA-free water) was added to the column, and the column incubated for 15 min at room temperature. The column was washed with 350 µL of RW1 and twice with 500 µL of RPE buffer (QIAGEN©), according to the manufacturer’s recommendations. The column was further centrifuged for 2 min at maximum speed (13000 rpm, 19000 g) to eliminate traces of buffers. The total RNA was collected in an RNAse-free Eppendorf tube after adding 25–40 µL of RNA-free water to the column, a 5-min incubation, and a centrifugation step for 1 min at 13000 rpm. Eluted RNA was stored at -80 °C with the addition of 0.4 µL of RNasin© to increase stability.

The concentration and the quality of RNA samples were assessed using an ND-1000 spectrophotometer (NanoDrop Technologies) and a Fragment Analyzer™ Automated CE System (Advanced Analytical). The final quality of the RNA was at least

roughly 7 RQN to ensure good cDNA library preparations and subsequent RNA-seq analysis (see Fig. S5).

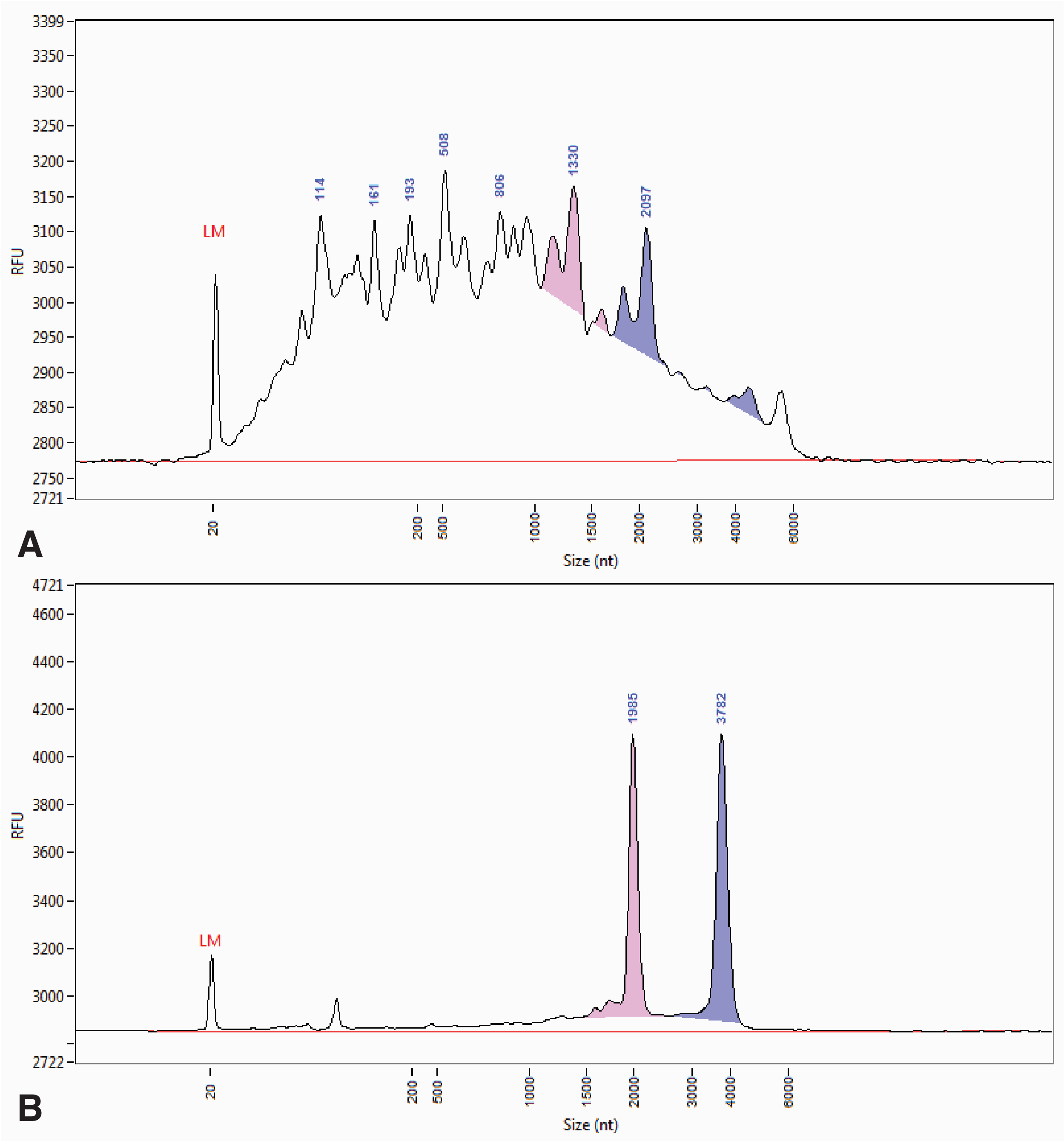

FIG S5 Quality of RNA extracted using the QIAGEN RNA extraction kit (A) and the protocol described in material and method section (B). The 18s rRNA and 28s rRNA absorbance peaks are shown in purple and blue, respectively.

SUPPLEMENTARY RESULTS

Correction of SUB10 and SED3 gene sequences

Arthroderma benhamiae Lau2354-2 genomic DNA was isolated from freshly growing mycelium using a Qiagen DNAeasy Plant mini Kit (Qiagen, Hilden, Germany). The region surrounding the predicted assembly error in ARB_06467 (SUB10) was amplified using primers 5'-GGGACTTCCGATGCCACATTACTGG-3' (forward) and 5'-CCTCTTGTGAACGTCGCTGGCCC-3' (reverse). The region surrounding the predicted error in ARB_04677 coding for the N-terminus of SED3 was amplified using primers 5'-TTCCTGAAGGATGGGTTCAGGGG-3' (forward) and 5'-TGTAAAGCTCTCGTAAACATCTTGG-3' (reverse). PCR products were sent to Microsynth AG (Balgach, Switzerland) for Sanger DNA sequencing. The new sequences confirmed the predictions of errors. Protein sequences for SUB10 and SED3 were updated accordingly and submitted to the UniProtKB database (accessions D4AQG0 and D4AK75).

Detailed functional characterization of the secretome

Proteins with proteolytic activity are overrepresented in the *A. benhamiae* secretome with no less than 76 proteins. Endoprotease families, such as subtilisins (S8),

deuterolysins (M35), and fungalysins (M36), are highly amplified (1). Half of them (38 proteins) have been identified in the MS data.

Sixty-nine predicted genes encode proteins that may be involved in carbohydrate/cell wall metabolism, including five putative chitinases (ARB_02187, ARB_00328, ARB_03514, ARB_05154, and ARB_07371). Twenty two among those have been confirmed by MS. In addition, eight genes, ARB_05157 (confirmed by MS), ARB_03442, ARB_00327, ARB_01155, ARB_03438, ARB_01488, and the two newly predicted ARBNEW_52 and ARBNEW_308, encode proteins containing LysM domains, involved in peptidoglycan-binding and found in bacterial virulence factors (2). It has been proposed that fungal LysM-containing secreted proteins have a role in sequestration of chitin oligosaccharides resulting from degradation of the cell walls, which can trigger host immunity, thus attenuating host defense (3). Cell walls of several pathogenic fungi indeed have been demonstrated to induce host inflammatory responses (4–6).

Fourteen proteins with putative lipolytic activity, such as the phospholipase C homologs ARB_04618, ARB_01728, and ARB_06966, or the phospholipase A homolog ARB_02001 were identified. Nine have been retrieved in the MS data.

Five genes, ARB_02157, ARB_01347, ARB_00194, ARB_07070, and ARB_08047, encode potential secreted ribonucleases. ARB_07070 is the *A. benhamiae* homolog of the major *Arthroderma fumigatus* allergen and cytotoxin Asp fI (7). This protein belongs to the mitogillin family containing highly specific ribonucleases that enzymatically inactivate the host ribosome by cleaving the 28S RNA of the large ribosomal subunit at

a single phosphodiester bond, thus inhibiting protein synthesis (8, 9). ARB_08047 belongs to the fungal guanyl-specific ribonuclease family of secreted proteins (10).

Four uncharacterized proteins, ARB_01017, ARB_01545, ARB_04403, and ARB_02741, contain fungal-specific eight cysteines extracellular membrane (CFEM) domains and may play a role as mediators in host–pathogen interactions (11). ARB_01932 and ARB_01183 belong to the thaumatin-like gene family and may act as allergens (see Discussion section).

Many allergens have been identified in other fungal species (12, 13). We retrieved 95 of these from the Allergome database (http://www.allergome.org/), searched for homologs in the *A. benhamiae* proteome and identified 46 gene products. Twenty one are predicted to be secreted (Table S2). In addition, searching for homologs using Blast against UniProtKB revealed ARB_02861 to be homologous to venom allergen Ves v 5 from the vespid wasps (14).

Focus on secreted proteases

Secreted proteases are among the largest functional group identified in the secretome or at the surface of the cell (22 are probably GPI-anchored). They also represent the best characterized functional group in *A. benhamiae* (15), but also in other dermatophytes such as *T. rubrum* (1, 16). Therefore we focused our attention on this specific functional group.

Nine predicted proteases belong to the A1 aspartic protease family (15). Aspartic proteases use aspartic acid residues in their active site to attack peptide bonds. They are very common in eukaryotes and have broad specificity.

Two gene products belong to the C40 peptidase superfamily, also called the N1pC/P60 superfamily, which includes several diverse groups of proteins in addition to the well-characterized P60-like proteins (16). The C40 peptidases have been shown to be involved in peptidoglycan hydrolysis. One of the *A. benhamiae* C40 proteases identified is a newly predicted ORF, ARBNEW_81, not present in the previous genome annotation (1).

Metalloproteases are well represented, with the identification of 29 members belonging to the M10B, M12B, M14, M19, M20, M28, M35, M36, and M43 families. Thirteen secreted metalloproteases have been being confirmed by mass spectrometry. The M36 family containing the fungalysins is the largest *A. benhamiae* metalloprotease family with eight members.

Finally, 35 identified secreted proteases are serine proteases belonging to the S1, S8, S9, S10, S28, S33, S41, and S53 families. Twenty have been confirmed by mass spectrometry. The S8 family containing the subtilisins has been previously shown to be expanded in dermatophytes (1). No fewer than 11 subtilisin-like proteases have been identified. Subtilisin-like serine proteases make up one of the most important allergen families. Many S8 family members have been identified as acting as allergens in fungi, such as in *Aspergillus* (17), *Penicillium* (17, 18), *Rhodotorula* (19), *Cladosporium* (20), *Curvularia* (21) and *Trichophyton* species (22).

The serine-carboxyl peptidase family S53 (also called the sedolisins) is very closely related to the subtilisins of the S8 family (23), and like for the subtilisins, the sedolisins are widespread among filamentous fungi (24). In *Aspergillus fumigatus*, secreted

sedolisins degrade large peptides from their N-terminus into tripeptides at acidic pH (25).

The *A. benhamiae* secretome also includes homologs of the very well-characterized dipeptidyl peptidases DPPIV and DPPV of the S9 family (26). DPPIV has been identified as an allergen in several dermatophytes, including *T. tonsurans* and *T. rubrum* (27).

Finally, the S10 serine-carboxypeptidase family is also well represented with 10 members, five of them having been confirmed by mass spectrometry. Secreted acidic serine carboxypeptidases of the S10 family have been well-characterized in *Aspergillus niger* (28, 29) and *Aspergillus oryzae* (30) as well as, more recently, in the pathogenic *A. fumigatus* and *T. rubrum* species (31).

REFERENCES

1. **Burmester A**, **Shelest E**, **Glöckner G**, **Heddergott C**, **Schindler S**, **Staib P**, **Heidel A**, **Felder M**, **Petzold A**, **Szafranski K**, **Feuermann M**, **Pedruzzi I**, **Priebe S**, **Groth M**, **Winkler R**, **Li W**, **Kniemeyer O**, **Schroeckh V**, **Hertweck C**, **Hube B**, **White TC**, **Platzer M**, **Guthke R**, **Heitman J**, **Wöstemeyer J**, **Zipfel PF**, **Monod M**, **Brakhage AA**. 2011. Comparative and functional genomics provide insights into the pathogenicity of dermatophytic fungi. Genome Biol **12**:R7.
2. **Buist G**, **Steen A**, **Kok J**, **Kuipers OP**. 2008. LysM, a widely distributed protein motif for binding to (peptido)glycans. Mol Microbiol **68**:838–847.
3. **de Jonge R**, **Thomma BPHJ**. 2009. Fungal LysM effectors: extinguishers of host immunity? Trends Microbiol **17**:151–157.

4. **Silva CL**, **Alves LM**, **Figueiredo F**. 1994. Involvement of cell wall glucans in the genesis and persistence of the inflammatory reaction caused by the fungus Paracoccidioides brasiliensis. Microbiol Read Engl **140 (Pt 5)**:1189–1194.

5. **Young S-H**, **Ostroff GR**, **Zeidler-Erdely PC**, **Roberts JR**, **Antonini JM**, **Castranova V**. 2007. A comparison of the pulmonary inflammatory potential of different components of yeast cell wall. J Toxicol Environ Health A **70**:1116–1124.

6. **Villegas LR**, **Kottom TJ**, **Limper AH**. 2012. Chitinases in Pneumocystis carinii pneumonia. Med Microbiol Immunol (Berl) **201**:337–348.

7. **Arruda LK**, **Platts-Mills TA**, **Fox JW**, **Chapman MD**. 1990. Aspergillus fumigatus allergen I, a major IgE-binding protein, is a member of the mitogillin family of cytotoxins. J Exp Med **172**:1529–1532.

8. **Lamy B**, **Moutaouakil M**, **Latge JP**, **Davies J**. 1991. Secretion of a potential virulence factor, a fungal ribonucleotoxin, during human aspergillosis infections. Mol Microbiol **5**:1811–1815.

9. **Kao R**, **Davies J**. 1999. Molecular dissection of mitogillin reveals that the fungal ribotoxins are a family of natural genetically engineered ribonucleases. J Biol Chem **274**:12576–12582.

10. **Yakovlev GI**, **Moiseyev GP**, **Bezborodova SI**, **Both V**, **Sevcik J**. 1992. A comparative study on the catalytic properties of guanyl-specific ribonucleases. Eur J Biochem FEBS **204**:187–190.

11. **Zhang Z-N**, **Wu Q-Y**, **Zhang G-Z**, **Zhu Y-Y**, **Murphy RW**, **Liu Z**, **Zou C-G**. 2015. Systematic analyses reveal uniqueness and origin of the CFEM domain in fungi. Sci Rep **5**:13032.

12. **Horner WE**, **Helbling A**, **Salvaggio JE**, **Lehrer SB**. 1995. Fungal allergens. Clin Microbiol Rev **8**:161–179.

13. **Crameri R**, **Garbani M**, **Rhyner C**, **Huitema C**. 2014. Fungi: the neglected allergenic sources. Allergy **69**:176–185.

14. **Henriksen A**, **King TP**, **Mirza O**, **Monsalve RI**, **Meno K**, **Ipsen H**, **Larsen JN**, **Gajhede M**, **Spangfort MD**. 2001. Major venom allergen of yellow jackets, Ves v 5: structural characterization of a pathogenesis-related protein superfamily. Proteins **45**:438–448.

15. **Szecsi PB**. 1992. The aspartic proteases. Scand J Clin Lab Investig Suppl **210**:5–22.

16. **Anantharaman V**, **Aravind L**. 2003. Evolutionary history, structural features and biochemical diversity of the NlpC/P60 superfamily of enzymes. Genome Biol **4**:R11.

17. **Shen HD**, **Tam MF**, **Chou H**, **Han SH**. 1999. The importance of serine proteinases as aeroallergens associated with asthma. Int Arch Allergy Immunol **119**:259–264.

18. **Shen HD**, **Lin WL**, **Tam MF**, **Wang SR**, **Tzean SS**, **Huang MH**, **Han SH**. 1999. Characterization of allergens from Penicillium oxalicum and P. notatum by immunoblotting and N-terminal amino acid sequence analysis. Clin Exp Allergy J Br Soc Allergy Clin Immunol **29**:642–651.

19. **Chou H**, **Tam MF**, **Lee S-S**, **Tai H-Y**, **Chang C-Y**, **Chou C-T**, **Shen H-D**. 2005. A vacuolar serine protease (Rho m 2) is a major allergen of Rhodotorula mucilaginosa and belongs to a class of highly conserved pan-fungal allergens. Int Arch Allergy Immunol **138**:134–141.

20. **Vishwanath S**. 1991. Antigenic relationships among the rickettsiae of the spotted fever and typhus groups. FEMS Microbiol Lett **65**:341–344.

21. **Tripathi P**, **Nair S**, **Singh BP**, **Arora N**. 2011. Molecular and immunological characterization of subtilisin like serine protease, a major allergen of Curvularia lunata. Immunobiology **216**:402–408.

22. **Jousson O**, **Léchenne B**, **Bontems O**, **Capoccia S**, **Mignon B**, **Barblan J**, **Quadroni M**, **Monod M**. 2004. Multiplication of an ancestral gene encoding secreted fungalysin preceded species differentiation in the dermatophytes Trichophyton and Microsporum. Microbiol Read Engl **150**:301–310.

23. **Wlodawer A**, **Li M**, **Gustchina A**, **Oyama H**, **Dunn BM**, **Oda K**. 2003. Structural and enzymatic properties of the sedolisin family of serine-carboxyl peptidases. Acta Biochim Pol **50**:81–102.

24. **Reichard U**, **Léchenne B**, **Asif AR**, **Streit F**, **Grouzmann E**, **Jousson O**, **Monod M**. 2006. Sedolisins, a new class of secreted proteases from Aspergillus fumigatus with endoprotease or tripeptidyl-peptidase activity at acidic pHs. Appl Environ Microbiol **72**:1739–1748.

25. **Sriranganadane D**, **Waridel P**, **Salamin K**, **Reichard U**, **Grouzmann E**, **Neuhaus J-M**, **Quadroni M**, **Monod M**. 2010. Aspergillus protein degradation pathways with different secreted protease sets at neutral and acidic pH. J Proteome Res **9**:3511–3519.

26. **Vermout S**, **Baldo A**, **Tabart J**, **Losson B**, **Mignon B**. 2008. Secreted dipeptidyl peptidases as potential virulence factors for Microsporum canis. FEMS Immunol Med Microbiol **54**:299–308.

27. **Woodfolk JA**, **Wheatley LM**, **Piyasena RV**, **Benjamin DC**, **Platts-Mills TA**. 1998. Trichophyton antigens associated with IgE antibodies and delayed type hypersensitivity. Sequence homology to two families of serine proteinases. J Biol Chem **273**:29489–29496.

28. **Dal Degan F**, **Ribadeau-Dumas B**, **Breddam K**. 1992. Purification and characterization of two serine carboxypeptidases from Aspergillus niger and their use in C-terminal sequencing of proteins and peptide synthesis. Appl Environ Microbiol **58**:2144–2152.

29. **Svendsen I**, **Dal Degan F**. 1998. The amino acid sequences of carboxypeptidases I and II from Aspergillus niger and their stability in the presence of divalent cations. Biochim Biophys Acta **1387**:369–377.

30. **Blinkovsky AM**, **Byun T**, **Brown KM**, **Golightly EJ**. 1999. Purification, characterization, and heterologous expression in Fusarium venenatum of a novel serine carboxypeptidase from Aspergillus oryzae. Appl Environ Microbiol **65**:3298–3303.

31. **Zaugg C**, **Jousson O**, **Léchenne B**, **Staib P**, **Monod M**. 2008. Trichophyton rubrum secreted and membrane-associated carboxypeptidases. Int J Med Microbiol IJMM **298**:669–682.
