# Supplementary figures and images for: RNA Sequencing-Based Genome Reannotation of the Dermatophyte Arthroderma benhamiae and Characterization of Its Secretome and Whole Gene Expression Profile during Infection
Source: mSystems. 2016 Aug 2;1(4):e00036-16. doi: 10.1128/mSystems.00036-16 (PMC5069957; doi:10.1128/mSystems.00036-16)

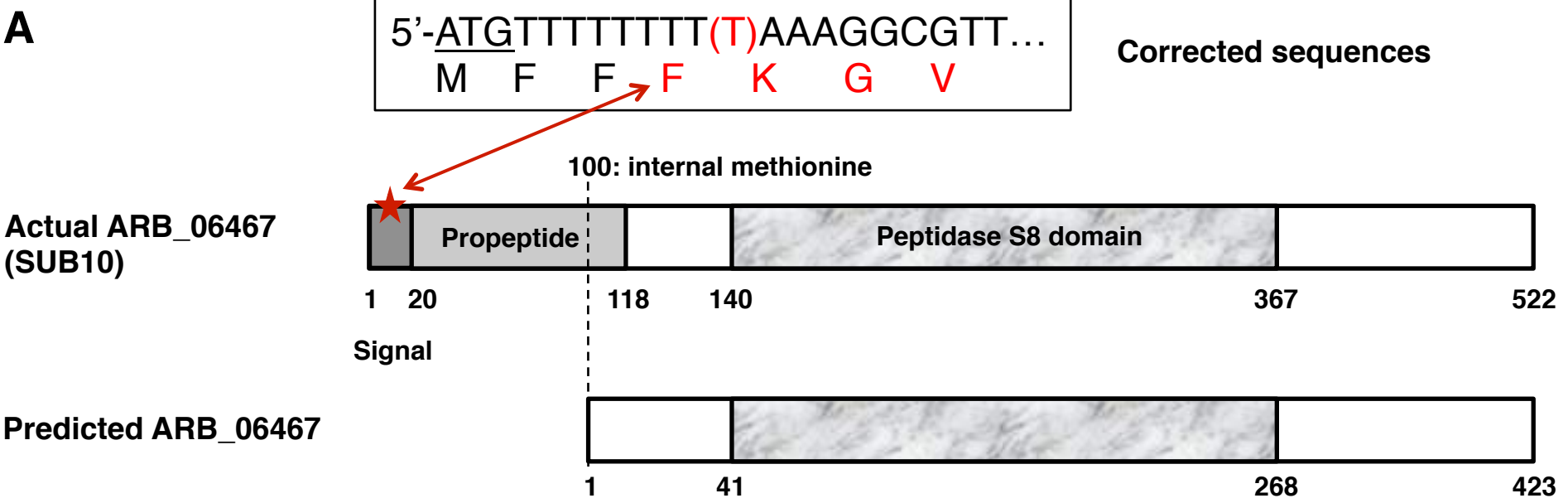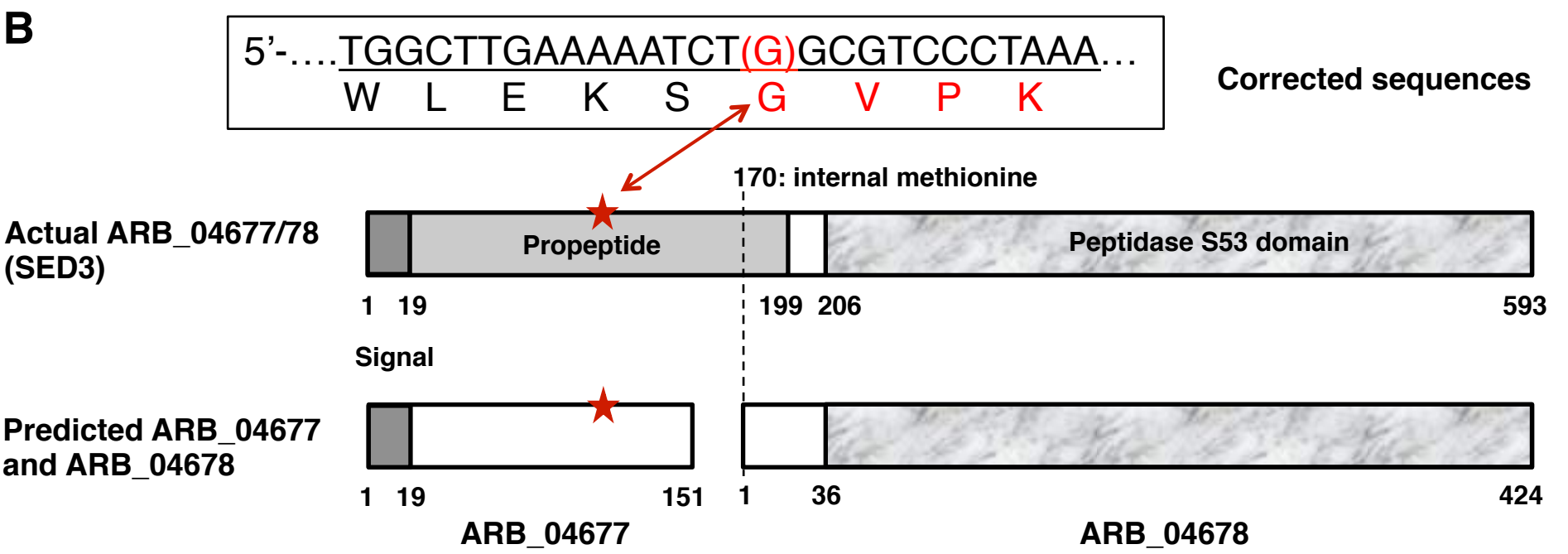

Supplement: Figure S1 [file sys004162042sf2.pdf]

Module-Contrast Correlations

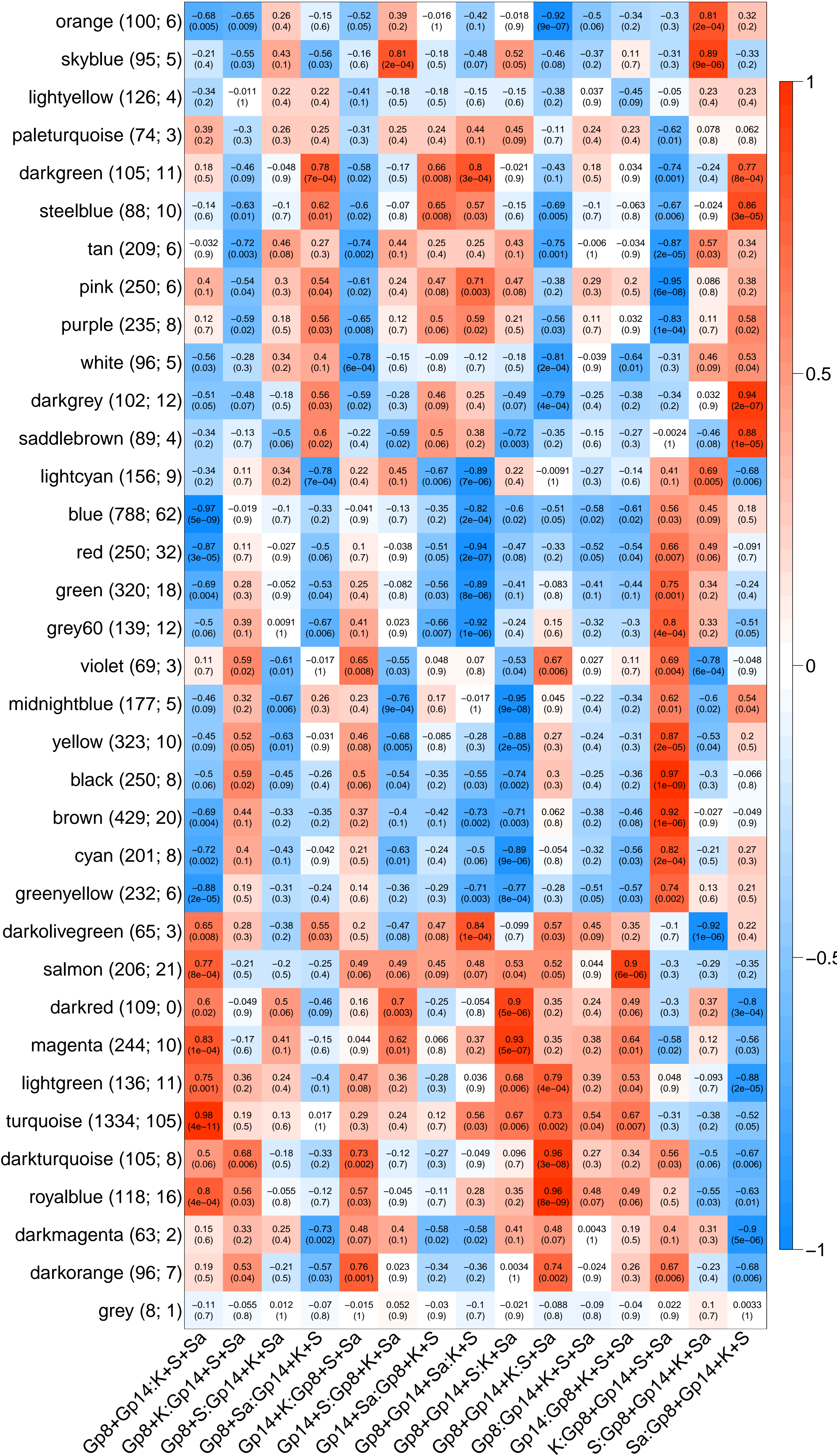

Supplement: Figure S2 [file sys004162042sf3.pdf]

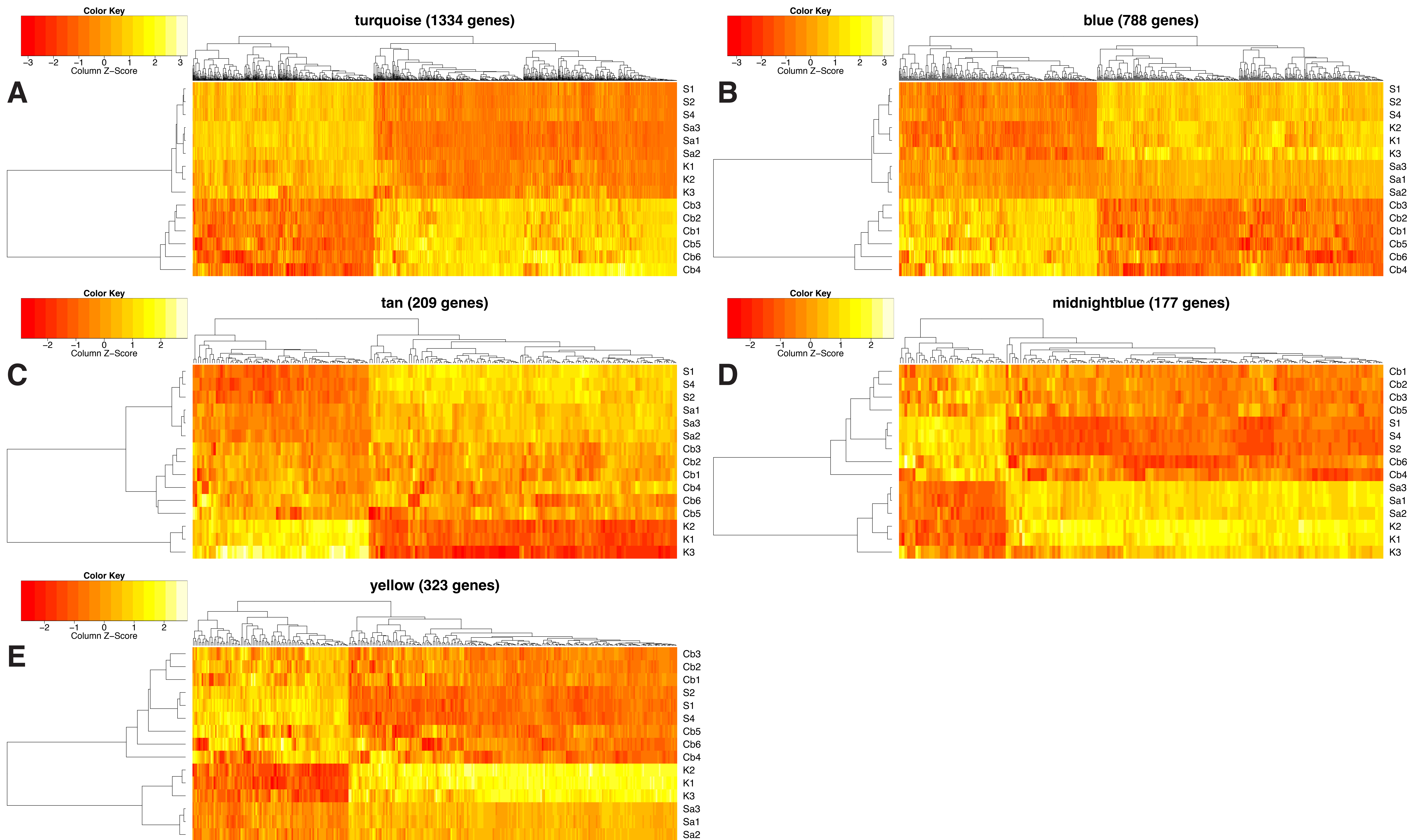

Supplement: Figure S3 [file sys004162042sf4.pdf]

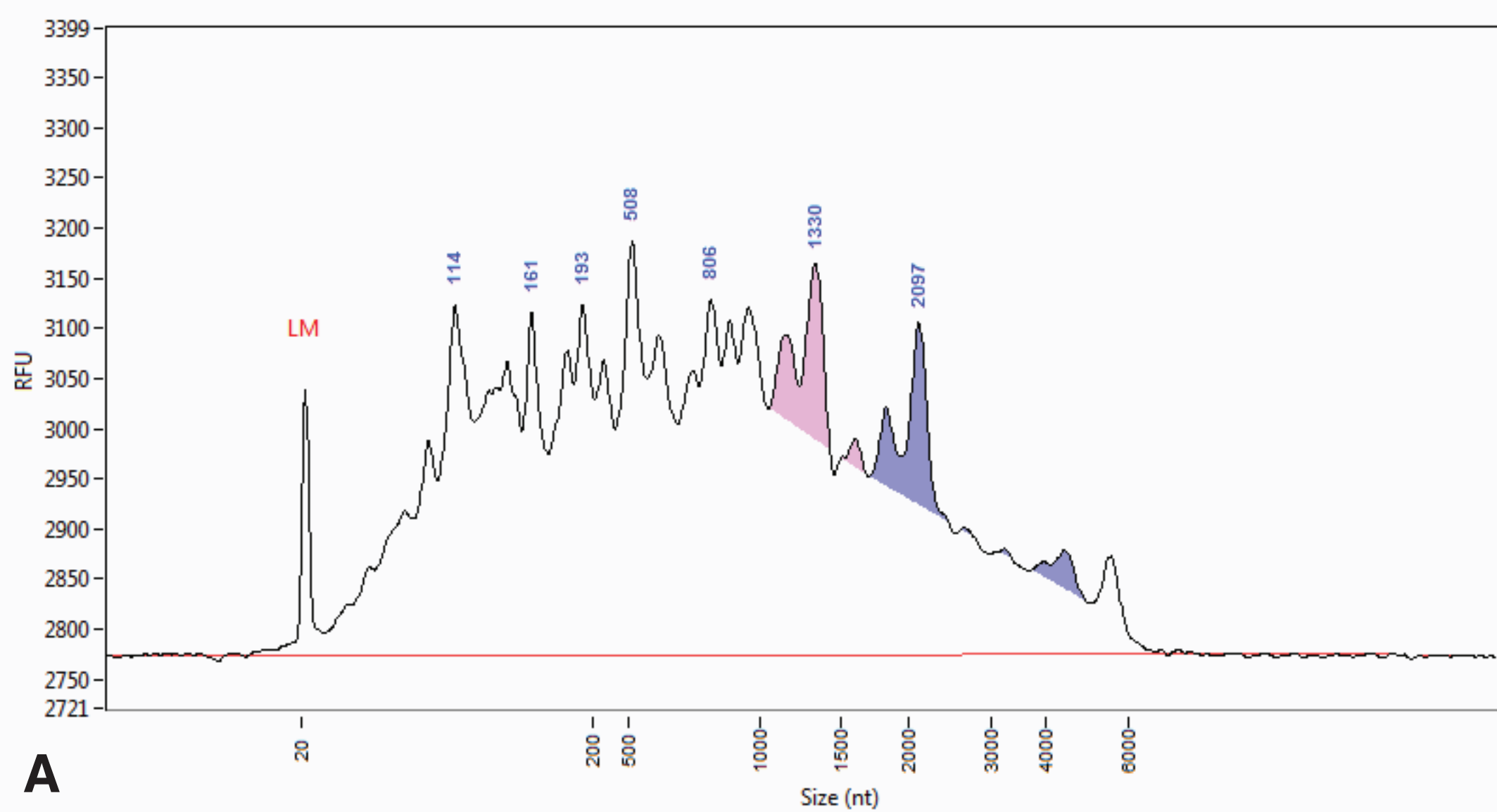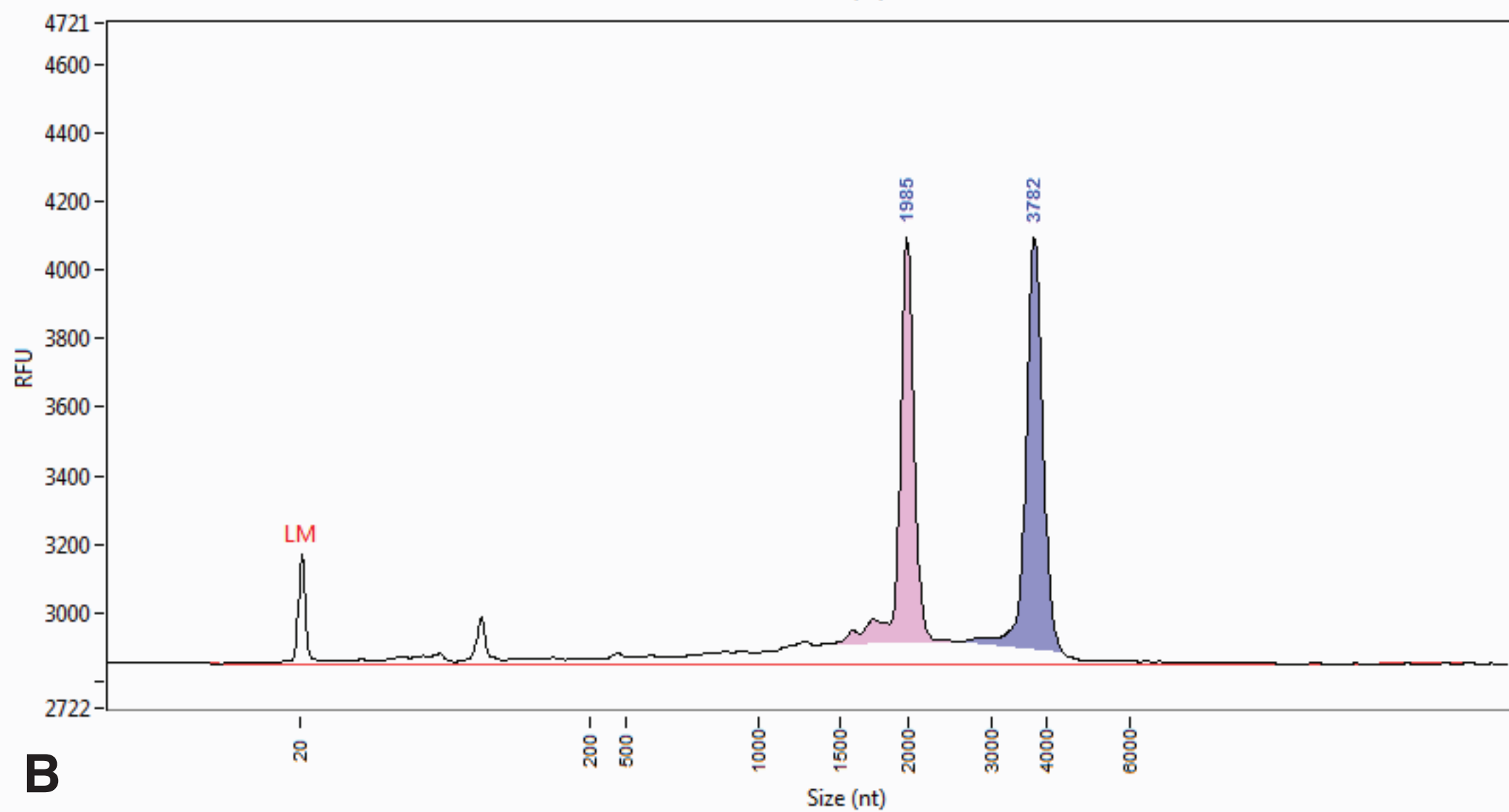

Supplement: Figure S5 [file sys004162042sf6.pdf]
