# Supplementary material for: RNA Sequencing-Based Genome Reannotation of the Dermatophyte Arthroderma benhamiae and Characterization of Its Secretome and Whole Gene Expression Profile during Infection
Source: mSystems. 2016 Aug 2;1(4):e00036-16. doi: 10.1128/mSystems.00036-16 (PMC5069957; doi:10.1128/mSystems.00036-16)
Supplement: Figure S4 [file sys004162042sf5.pdf]

	Gp8	K	
Subtilisin−like protease SUB6 : ARB_05307	5036.99	14610.88	ARB_00701 : Subtilisin−like protease SUB3
Neutral protease 2 homolog : ARB_04336	1156.36	8947.14	ARB_01032 : Subtilisin−like protease SUB4
**Aspartic−type endopeptidase PEP2 : ARB_02919**	843.45	7272.66	ARB_02803 : Uncharacterized protein
Subtilisin−like protease SUB10 : ARB_06467	791.13	3064.29	ARB_03568 : Leucine aminopeptidase LAP1
Subtilisin−like protease SUB8 : ARB_00777	642.2	2133.1	ARB_07026_07027 : Metallocarboxypeptidase MCPA
Carboxypeptidase Y homolog A CPYA : ARB_01491	528.34	2112.63	ARB_04170 : Aspartic−type endopeptidase OPSB
Probable serine carboxypeptidase : ARB_06414	459.6	1796.25	ARB_05317 : Probable metaloproteinase
Subtilisin−like protease SUB1 : ARB_04944	422.02	1792.08	ARB_00494 : Leucine aminopeptidase LAP2
Peptidase S41 family protein : ARB_02997	181.69	1370.48	ARB_05085 : Extracellular metalloprotease MEP3 (fungalysin)
Probable glutamate carboxypeptidase : ARB_02390	150.69	792.98	ARB_06110 : Dipeptidyl peptidase 4 DPPIV
Carboxypeptidase S1 homolog A SCPA : ARB_04046	133.52	760.65	ARB_06651 : Dipeptidyl peptidase 5 DPPV
Putative metallocarboxypeptidase ECM14 : ARB_04942	125.9	691.09	**ARB_02919 : Aspartic−type endopeptidase PEP2**
